# Do inhabitants profit from integrating a public health focus in urban renewal programmes? A Dutch case study

**DOI:** 10.1371/journal.pone.0270367

**Published:** 2022-06-24

**Authors:** Annemarie Ruijsbroek, Albert Wong, Frank den Hertog, Mariël Droomers, Carolien van den Brink, Anton E. Kunst, Hans A. M. van Oers, Karien Stronks

**Affiliations:** 1 National Institute for Public Health and the Environment (RIVM), Bilthoven, The Netherlands; 2 Department of Public Health, Municipality of Utrecht, Utrecht, The Netherlands; 3 Department of Public and Occupational Health, Amsterdam Public Health Research Institute, Amsterdam UMC, University of Amsterdam, Amsterdam, The Netherlands; 4 Tranzo, Faculty of Social Sciences, University of Tilburg, Tilburg, The Netherlands; Flinders University, AUSTRALIA

## Abstract

**Background:**

Urban renewal traditionally involves policy sectors such as housing, transport, and employment, which potentially can enhance the health of residents living in deprived areas. Additional involvement of the public health sector might increase the health impact of these urban renewal activities. This study evaluates the health impact of an additional focus on health, under the heading of Healthy District Experiments (HDE), within districts where an urban renewal programme was carried out.

**Methods:**

We evaluated changes in health outcomes before the start of the HDE and after implementation, and compared these changes with health changes in control areas, e.g. districts from the urban renewal programme where no additional HDE was implemented. Additionally, we gathered information on the content of the experiments to determine what types of activities have been implemented.

**Results:**

The additional activities from the HDE were mostly aimed at strengthening the health care in the districts and at promoting physical activity. When we compared the prevalence in general health, mental health, overweight, obesity, smoking, and physical activity during the study period between the HDE districts and control districts, we found no significant differences in the rate of change. The study is limited by a small sample size and the cross-sectional nature of the data. These and other limitations are discussed.

**Conclusion:**

We found no evidence for a beneficial health impact of the activities that were initiated with a specific focus on health, within a Dutch urban renewal programme. Specific attention for network management and the integration of such activities in the wider programme, as well as an allocated budget might be needed in order to sort a health impact.

## Introduction

To tackle the root causes of poor health and socioeconomic health inequalities, it is important to address the social determinants of health, i.e. the circumstances in which people are born, grow, live, work, and age [1–3]. Improving the living circumstances in deprived neighbourhoods is one possible way to intervene on these social determinants of health with the aim to tackle socioeconomic health inequalities.

Urban renewal projects or area-based initiatives (ABI´s) pre-eminently aim to ameliorate the poor living circumstances in deprived neighbourhoods, such as the quality of housing, transport, the physical and social environment, employment, and public services. The Netherlands has a long history in urban renewal. In the seventies, the focus was put primarily on improving the physical quality of (pre-war) housing, which broadened in the mid-eighties and nineties towards addressing social problems in deprived neighbourhoods and combatting (further) area segregation [4]. In the most recent, nation-wide urban renewal programme from 2007, attention for the personal situation of residents, such as their job situation, has been part of the approach as well [5].

The activities that are conducted under the heading of urban renewal are potentially important means of improving health and reducing health inequalities [6], but so far, studies found limited empirical evidence that urban renewal programmes improve the health of residents [7–12].

Perhaps, the lack of an explicit focus on the health of the population can explain the lack of impact. With some exceptions, such as the Health Action Zones in the U.K. [13], urban renewal projects are traditionally organized and implemented outside the sphere of influence of the public health sector, with no explicit focus on improving populations’ health [4]. Instead, an improvement in health is considered the outcome of efforts in other policy areas, such as antipoverty policies and urban planning. Involvement of the public health sector might lead to more guidance towards activities from which a health impact could be expected. More specifically, more involvement of the public health sector could increase the effectiveness of the implemented interventions in terms of health enhancement, hence potentially increasing the health benefits of urban renewal programmes.

In the current study, we explore the health impact of the involvement of public health organisations in urban regeneration programmes by studying the Dutch Healthy District Experiment (HDE). The HDE is an area-based initiative that was initiated by the Dutch Ministry of Health, Welfare and Sport in 2008. It aimed at improving the health of residents in deprived areas and was carried out within the context of the Dutch District Approach, which was a large-scale urban renewal programme that ran from 2007 to 2012 [14]. In this urban renewal programme, around 5 billion euros was spent to ameliorate problems with employment, education, housing and the physical environment, safety, and social cohesion in the 40 most deprived urban districts situated in 18 large Dutch cities, all with an urban character [14].

Improving the health of residents was not an explicit aim of Dutch District Approach. The HDE tried to fill this gap. The experiment had the explicit goal to include a public health perspective in the renewal of the deprived districts and was implemented in a subset of target districts within the urban renewal programme. The overall aim at the start of the experiment was to improve the health of the residents structurally within ten years’ time [15].

In this study, we compare the health trends in the HDE target districts with the trends in the districts that were also part of the urban renewal programme, but did not participate in the HDE, that is, had no explicit health goal (non-HDE target districts). We also explore the content of the experiments to determine what types of activities have been implemented. Evaluating the HDE´s can provide insight in whether the involvement of the public health domain could increase the effectiveness of urban renewal programmes in terms of health enhancement.

## Methods

### The HDE intervention

In 2008, the Dutch Ministry of Health, Welfare and Sport in cooperation with the former Ministry of Housing, Districts and Integration, initiated the ‘Healthy District Experiments’ (HDE). The HDE was implemented in 19 of the 40 target districts of the Dutch District Approach and comprised of activities to improve the health of the local population, such as creating small scale sport fields and playgrounds or providing health information in foreign languages. These activities were an addition to the activities on the five policy themes of the District Approach (i.e. employment, education, housing and the physical environment, safety, and social cohesion). The implementation of the HDE started between 2009 and 2011. Even though the initial aim was to improve the health in the HDE districts in a period of ten years, the experiments stopped in February 2014, when the national government implemented a new programme for tackling health inequalities at the local level, as part of the new National Prevention Program.

The overall aim of the Ministry of Health, Welfare and Sport was to improve the health of the residents of the deprived districts within ten years’ time [15]. They aimed at bringing the health of the residents in these target areas closer to the level of the rest of the municipality [15].

At the start of the experiments, the Ministry provided a framework for the municipalities, based on a Dutch conceptual model on reducing socio-economic health inequalities [16]. The framework consists of four points of action: improving the participation in society (work, schooling) of residents with health problems, improving the socio-economic position of residents, improving the living environment and the health-related behaviour of residents, and strengthening health care in the neighbourhood. The municipalities were encouraged by the Ministry to take up an integral approach and combine action points in their experiments [15]. They were given autonomy in choosing the action points, based on the specific health problems in the HDE-districts, as well as in how they wanted to give substance to the action points, in terms of which activities to implement and the process chosen.

The Ministry of Health, Welfare and Sport supported the participating municipalities with the development and implementation of the HDE’s. The Ministry created a supporting infrastructure, which consisted of so-called inspiration sessions and one-on-one consultation with the Ministry. The inspiration sessions were organised four times a year between 2008 and 2014. At these gatherings, the HDE policymakers of the participating municipalities could meet each other and learn from each other’s experiences. The consultation sessions were held with the municipalities at least once a year to offer them expert advice and to discuss the progress of the experiments. There was no funding available from the Ministry for the implementation of the Healthy District Experiments at the local level.

Application for participation in a Healthy District Experiment was on voluntary basis. At the start of the overall urban renewal programme, nine municipalities had mentioned that they planned to address health in some way in the programme. These municipalities were approached by the Ministry to participate in the HDE. Finding more municipalities willing to participate in the experiments was challenging. Eventually, when the former Ministry of Housing, Districts and Integration actively approached the other municipalities to get involved in the experiment, four additional municipalities joined the initiative, bringing the total to 13 municipalities (covering 19 HDE-districts).

### Study population

Data on the health of the residents in the districts were obtained from nationwide yearly cross-sectional health data from the Dutch Health Survey (HIS) 2003–2014, from Statistics Netherlands. Respondents of this survey are of all ages, living in private households in the Netherlands. According to Dutch law (Wet Medisch Wetenschappelijk Onderzoek met mensen), formal approval (e.g., from a medical ethics committee) was not required as this study relied on secondary anonymized data collection in the context of performing statutory tasks. Data were provided by Statistics Netherlands in a way that no individual person can be identified. Details on data protection issues are described on the website of Statistics Netherlands: Privacy (cbs.nl). For details on the data collection of the Dutch Health Survey see [10].

For the current study, we selected data from respondents who lived in the target districts or the selected control and were at least 18 years old on January 1 ^st^ 2008.

### Matching on districts and individuals

The 40 target districts from the Dutch District Approach were divided into districts where Healthy District Experiments (HDE’s) had been implemented (18 HDE target districts) versus not (21 non-HDE target districts). One HDE target district was labelled as missing, because we were unable to interview a professional with knowledge of the implemented activities within the experiment. The HDE-target districts and non-HDE target districts were matched by means of 1:1 Nearest Neighbor Matching on area characteristics (e.g. the percentage of old housing and social housing units, the percentage of inhabitants having encountered vandalism; 13 indicators in total, see S1 File for the full list of variables). This means that each HDE-target district was matched at postal code level to a non-HDE target district that was most similar in terms of these area characteristics. Target districts could encompass several postal codes. The area characteristics were measured at January 1^st^ 2008, i.e. just prior to the start of the implementation of the District Approach.

After the matching procedure at the area-level, we selected all individuals from the selected districts and stratified them based on their individual characteristics. Following [10], we stratified per outcome on the most relevant individual characteristics for that outcome, because full stratification on all available characteristics (age, sex, education, income, ethnicity and household composition) led to very small strata. In total, we had between 990 and 3,048 respondents in the analysis, depending on the outcome. For details on the matching procedure, see S1 File.

Health data were divided into a pre-intervention period (2003-mid 2008), and two intervention periods: early intervention (mid 2008–2011) and late intervention (2012-2013/’14). Most health interventions of the Healthy District Experiments were implemented a few years after the start of the urban renewal programme. Therefore, we were interested in the health developments in the late intervention period in order to detect health improvements caused by the Healthy District Experiments. In the analyses, we compared the pre-intervention period with the late intervention period.

### Health outcomes

We included the same health outcomes as used in a previous evaluation of the Dutch District Approach, i.e. *general health* (good general health versus less than good general health), *mental health* (fair or good mental health versus less than fair or good mental health), *leisure-time walking* ("inactive” (no minutes per week leisure-time walking) versus”active” (any minutes per week leisure-time walking)), *leisure-time cycling* (“inactive” (no minutes per week leisure-time cycling) versus”active” (any minutes per week leisure-time cycling)), *sport participation* (“inactive” (no minutes per week sport participation) versus”active” (any minutes per week sport participation)), *smoking* (yes/no), *overweight* (yes/no) and *obesity* (yes/no). The health outcome overweight also includes obesity. For details on the measurement of the health outcomes, see [10].

For general health, smoking, overweight and obesity, the pre-intervention period included the years 2003 to mid-2008 and the late intervention period the years 2012 to 2014. For mental health and physical activity, the pre-intervention period included the years 2004 to mid-2008 and the late intervention period the years 2012 and 2013, due to limitations in data availability.

### Content of the HDE

To gain insight in what kind of activities were implemented in the HDE-target districts, we held interviews with local professionals involved in the Healthy Districts Experiments. In total, 16 interviews were held between July 2015 and September 2016. We used a standardized questionnaire to collect information about the content of the experiment, duration, and whether a problem analysis was conducted beforehand. In addition, we interviewed the policymaker from the Ministry of Health, Welfare and Sport who was actively involved in the organisation of the HDE intervention at the national level to collect background information about the HDE (e.g. the reason for starting the HDE’s, the type of support the Ministry provided).

We used the information from the interviews with local professionals to get an overview of the type of activities that were implemented in the HDE target districts. We structured the activities using the four points of action the Ministry of Health had proposed beforehand. We used this information to interpret the findings from the analyses.

### Analysis

First, we calculated changes between pre- and late intervention period in the prevalence of good general health, fair or good mental health, physical activity (leisure-time walking, leisure-time cycling, and sport participation), smoking, overweight, and obesity in the HDE target districts and the non-HDE target districts. Next, the health trends in HDE target districts were compared with those in non-HDE target districts.

We estimated a stratum-specific Difference-in-Difference (DiD), which is defined as the change over time in the mean outcome in HDE target districts subtracted with the change in control districts (i.e. non-HDE target districts). The change is measured by subtracting the mean outcome in the late intervention period with the pre-intervention period. The difference in outcome for the control group served as a way to control for unmeasured confounding (e.g. general trends in health-related behaviour or health) under the assumption that this affected the HDE and control districts similarly. The DiD is estimated through a linear regression model in which the outcome was estimated as a function of a period indicator (late intervention period compared to the pre-intervention period), intervention indicator (treatment group, i.e. the HDE target districts, compared to the reference group, i.e. non-HDE target district), and the interaction between the period and treatment indicator (which reflects the actual DiD).

Finally, an overall intervention effect was obtained by pooling over all stratum-specific treatment effects by taking weighted averages over the mean and variances. The strategy follows the one used by [10]. R software, MatchIt package and own programming, was used for the quantitative analysis. Further detail of the statistical analysis can be found in S1 File.

## Results

### Implemented activities in the HDE target districts

In 14 of the 18 HDE target districts, a problem analysis was conducted at the start of the experiment to assess the health problems most prevalent in the targeted areas. Of the four districts that did not perform this exercise, one stated that the health problems were already known, one did an analysis but specifically on the problems in the cooperation between health professionals, one conducted the problem analyses later on in the experiment, and for one the reason was unknown.

Table 1 gives a general overview of the content of the HDE programme. Of the four points of action the Ministry of Health, Welfare and Sport had formulated for the municipalities (i.e. participation, socio-economic position, living environment/behaviour, and health care facilities), most HDE’s focussed on strengthening the health care in the neighbourhood (11 HDE’s), followed by improving the living environment and the health-related behaviour of residents (9 HDE’s) and improving participation in society (8 HDE’s). Activities to improve the socio-economic position of residents were implemented the least (Table 1). Even though the Ministry encouraged the municipalities to take up more than one action point, almost half of the HDE’s focussed on a single point of action. Exceptions were the HDE’s in the larger cities, where activities from several points of action were implemented, though often still with a focus on one action point in particular.

**Table 1:** Examples of implemented activities per point of action in the HDE target district.

Point of action	Number of HDE´s focussing on this strategy	Approaches/aims within the strategy	Examples of activities that have implemented in the different HDE-target districts
Improving the participation in society of residents with health problems	8	Tackling health obstacles that hinder residents to participate in society; Strengthening neighbourhood social capital	Initiatives to activate people who are long-term unemployed by addressing—amongst others persistent (mental) health issues Installation of neighbourhood coaches to help vulnerable residents (e.g. migrants, multi-problem families) to participate more in society Initiatives to prevent school delay of students because of health-related school absence Health brokers who initiate activities together with the residents
Improving the socio-economic position of residents	2	Leading unemployed residents to work, Preventing school dropout	Fit4work: reintegration of people with mental health problems into the workforce Creative Urbans: youth talent development program
Improving the living environment and the health-related behaviour of residents	9	Improving the living environment in such way that it promotes health and supports a healthy lifestyle Implementing activities to promote a healthy lifestyle	“Sport gardens” and green playgrounds to promote physical activity in public spaces Small scale sport fields and playgrounds (Cruyff courts, Krajicek playgrounds) Improving and implementing green areas for active use and to facilitate social contact Neighbourhood or school vegetable gardens JOGG: implementing an integrated approach against youth obesity (based on EPODE approach) Sports projects for children Sport festivals Buddy projects where residents are trained to support neighbours who want to stop smoking Training interventions to increase people’s resilience, improve coping strategies
Strengthening health care in the neighbourhood	11	Strengthening the cooperation between health professionals working in the HDE target districts; Implementing activities to increase the accessibility of health care facilities	Organising meetings between health professionals active in the HDE-districts General practitioner consultations at schools, Providing health care for illegal immigrants, Opening a new health centre in the neighbourhood Providing health information in foreign languages (*Voorlichting in Eigen Taal en Cultuur)* Appointing health care consultants with migrant backgrounds Health checks for specific target groups (elderly, migrant women) Training courses for health professionals about conducting motivating conversations with residents with overweight Tailor-made physical activity programmes for inactive patients (subscribed by the GP) Appointing neighbourhood nurses to serve as a connection between residents with care needs and health care/welfare organisations Integrated approach addressing psychological and poverty issues among the elderly Supporting voluntary caregivers

Within one point of action, a great variety of activities has been initiated. The municipalities themselves decided what kind of activities they wanted to implement. Table 1 provides examples of activities that were implemented in the experiments within each point of action. All HDE´s except one, implemented activities to promote physical activity (e.g. improving and implementing green areas for active use, organising sport activities for children). Activities that addressed other health-related behaviours, i.e. smoking and alcohol consumption, were implemented in about one third of the HDE’s. Improving people’s resilience and reducing mental health problems were also implemented in about one third of the HDE’s. Since strengthening the health care in the neighbourhood was the most important point of action, many activities addressed health care issues, such as tackling language barriers of migrants, improving the cooperation between healthcare professionals in the neighbourhood, improving access to health care for residents with care needs, etc. (Table 1).

### HDE impact: Trends in health and health–related outcomes during the study period

Table 2 compares the characteristics of the residents of the HDE target districts with residents of the non-HDE target districts. Residents of the HDE target districts were similar to the residents of the non-HDE target districts in terms of sex, but were more often from non-Dutch, non-Western origin, with lower income, and with a lower education. The largest differences in the population characteristics between the HDE and non-HDE districts were found for ethnicity, with around 10 percent points higher percentage of residents from non-Dutch, non-Western origin in the HDE districts compared to the non-HDE districts (Table 2).

**Table 2:** Characteristics of the study population^a^.

	Healthy District Experiment within Dutch District Approach (HDE)		Only Dutch District Approach (non-HDE)	
	Pre-intervention	Late intervention	Pre-intervention	Late intervention
**N respondents**	**957**	**335**	**1345**	**565**
**Sex (% female)**	51.7	55.8	53.3	53.5
**Age (%)**				
18–35 years	38.3	35.5	43.3	39.1
35–55 years	30.9	33.7	29.1	35.8
55 years and older	30.7	30.7	27.7	25.1
**Household composition (%)**				
Single with/without child(ren)	37.8	36.7	40.2	41.2
Couples with/without child(ren), with others	62.2	63.3	59.8	58.8
**Education (%)**				
Primary, secondary	89.1	72.6	80.1	66.5
Tertiary	10.9	27.4	19.9	33.5
**Ethnicity (%)**				
Ethnic Dutch, non-Dutch—Western	59.6	62.8	68.5	71.4
Non-Dutch, non-Western	40.4	37.2	31.5	28.6
**Household income (%)**				
First tertile (> €16,315)	42.5	35.5	36.8	32.0
Second tertile (€16,315 – €23,563)	34.7	29.8	32.3	37.4
Third tertile (> €23,563)	22.8	34.7	30.9	30.6

^a^ Characteristics represent average values over the period 2003 to 2014.

Table 3 compares the changes in health and health-related behaviour between the HDE-target districts and the non-HDE target districts. In the HDE target districts, leisure time cycling increased between the pre-intervention and late intervention period (41.9% and 45.8% respectively), but this was not a statistically significant change. The other outcomes either remained stable over time (mental health, smoking, sport participation) or worsened (general health, overweight, obesity, leisure time walking) between the pre-intervention and late intervention period in the HDE target districts. The change in obesity was borderline significant (p-value 0.05), the other changes were not statistically significant. In the non-HDE target districts, leisure time cycling increased as well between the pre-intervention and late intervention period (from 42.7% to 47.4%), although not statistically significant. Neither were the other changes in prevalence in the non-HDE target districts (the increase in fair or good mental health and walking in leisure time, the decrease in smoking prevalence, and the increase in overweight and obesity between the pre-intervention and late intervention period). Sport participation and general health remained stable over time. The somewhat different trends between the HDE and non-HDE target districts did not result in significant impact estimates.

**Table 3:** Comparison of health and health-related behaviour between 2003-mid 2008 and 2012–2014 in 18 target districts with an additional health goal and 21 districts without an additional health goal.

		Healthy District Experiment within Dutch District Approach (HDE)			Only Dutch District Approach district (non-HDE)				
		Pre-intervention	Late intervention		Pre-intervention	Late intervention		Late vs pre HDE—Late vs pre non-HDE ^a^	
		*2003-mid 2008*	*2012–2014*		*2003-mid 2008*	*2012–2014*			
	*N* ^j^	*%*	*%*	p-value	*%*	*%*	p-value	DiD (C.I.)^k^	p-value
Good general health^b^	2544	66.7	60.0	0.07	66.0	65.3	0.85	-6.0 (-15.9;3.9)	0.24
Fairly or good mental health^c^	1148	80.9	81.2	0.95	83.5	89.7	0.17	-5.9 (-18.8;6.9)	0.37
Overweight^d^	2279	52.2	56.7	0.28	49.6	54.3	0.25	-0.2 (-11.7;11.3)	0.97
Obesity^e^	2279	13.6	19.7	0.05	14.5	16.5	0.51	4.1 (-4.4;12.6)	0.35
Smoking^f^	3048	31.2	31.2	0.99	37.0	31.8	0.19	5.1 (-6.1;16.4)	0.37
Leisure-time walking^g^	1371	68.8	62.6	0.27	62.7	65.8	0.57	-9.3 (-24.4;5.9)	0.23
Leisure-time cycling^h^	1275	41.9	45.8	0.51	42.7	47.4	0.44	-0,8 (-17.8;16.1)	0.93
Sports participation^i^	990	37.4	38,5	0.86	36.0	36.4	0.95	0.8 (-17.1;18.7)	0.93

^a^ Reference category;

^b^ adjusted for age, household income;

^c^ adjusted for age, sex, household income;

^d^ adjusted for age, education;

^e^ adjusted for age, education;

^f^ adjusted for age, sex, education;

^g^ adjusted for age;

^h^ adjusted for age, education, ethnicity;

^i^ adjusted for age, education, household income, ethnicity;

^j^ The n is the sum of all six groups used in the analysis;

^k^ Difference in Difference (Confidence Intervals).

## Discussion

We investigated whether the additional public health focus in an urban regeneration programme resulted in a positive impact of the programme on the health of residents. We found no evidence for a positive health impact of these experiments that were implemented on top of the Dutch District Approach-activities that were implemented by other policy domains, such as housing and social benefit.

### Methodological considerations

Before we discuss the findings, several methodological limitations should be mentioned. The interviews with the local professionals who had worked on the HDE’s were held more than over a year (in a few cases more than two years) after the end of the experiments. This led to recall problems among several of the professionals. In addition, for several HDE’s it was difficult to find the appropriate local expert for the interview, because of job changes. As a result, the quality of the information and the amount of detail on the implemented activities differed considerably between the HDE’s, making a comprehensive inventory of the content, magnitude, reach, costs, participation, etc. of the HDE’s, which was originally our plan, unfeasible.

Although we used large-scale nationwide datasets, the relatively small numbers of respondents available for the analysis, as a result of the small number of HDE districts, limited the precision of our impact estimates.

Since we used repeated cross-sectional data, we could not account for migration flows in- and out of the target and control districts, which could lead to an under- or overestimation of the health impact of area-based interventions [7,8,17,18]. In order to find that migration might bias our finding, migration flows need to differ between the HDE-target and non-HDE target districts. We can think of no reason why this would be the case. Moreover, in a previous study, we found no indications that selective migration biased the health impact assessment of the Dutch District Approach [19]. In this study, we found no substantial increase or decrease of the in- and outflow of residents during the urban regeneration program. Also, the in and out migrants did not differ in health substantially. Therefore, we do not expect that migration has impacted our results.

The characteristics of the residents in the HDE district differed from that of the non-HDE district and control district residents, especially in case of ethnicity. We addressed this limitation by stratifying our analysis by these characteristics in order to address the potential impact on the results (see the S1 File for further information on the stratification).

Municipalities themselves decided whether they wanted to participate in the experiment. This self-selection could have introduced bias in the experiment. Since participation was on voluntary basis and without extra funding, arguably the municipalities most motivated to address health problems signed up for the experiment. Bias by self-selection could then have resulted in an overestimation of the effect. Since no effect was found, this potential bias is not relevant here.

Several of the activities that were labelled as Healthy District Experiment-activities, have been implemented in other, non-HDE districts as well, such as Fit4work (aimed at addressing mental health problems in unemployed adults). This may have diluted the effect of the experiments and resulted in an underestimation of the health impact of the HDE´s.

Finally, this study cannot make statements about the impact of the HDE activities on the health of the younger population, since the data comprised the adult population only (18+ years). A substantial part of the HDE interventions focussed on the youth, which means we could have missed some of the health impacts of the HDE with this study.

### Discussion of the findings

The explicit health goal of the HDE’s and the involvement of the public health sector in the urban renewal programme has led to new activities, mainly in the field of health care. However, we found no noticeable health impact in our study. The lack of impact might be due to the focus on health *care*, and, consequently, the fact that these experiments barely addressed health determinants outside the health sector, such as housing, work situation, and education.

Perhaps the involvement of the public health sector in the development of activities tackling these social determinants of health would have resulted in a more noticeable impact on public health in the targeted districts. Instead, the implementation of the HDE’s started two or even more years after the start of the District Approach, meaning that a fully integrated approach with solid involvement of the public health sector did not occur, at least not from the start. To be able to contribute to population health, the involvement of actors from multiple policy sectors in an integrated approach is required [20]. This lack of a comprehensive intersectoral approach has arguably reduced the potential health impact of the initiative. Another Dutch study [21] showed that network diversity (over 50% of the actors outside public health sector) and intensive network management are important for intersectoral policy networks to be able to implement different policy instruments necessary to address the social determinants of health. These conditions were not consistently met here.

Most HDE’s started with a problem analysis that described the health problems in the target district, though not always with a thorough analysis of how these are linked to underlying causes and mechanisms. When an initiative starts from a problem analysis that includes the personal determinants of health and health behaviour, but not so much how this is related to environmental and social determinants of health and health behaviour, it is difficult for intersectoral policy networks to develop and reach consensus on intersectoral solutions [21]. The activities that were implemented were often based on the needs expressed by residents or on opportunities arisen within the organisation. The lack of a more focussed, intersectoral action plan might be a reason for the HDE not having a discernible health impact at the district level.

Furthermore, there was no additional funding from the national government available for the implementation of the HDE’s. Although we were not able to get a good insight in the expenses that were made in each HDE, it is arguable that the lack of extra funding for this experiment has hampered the implementation in such way that it was not able to achieve health improvements in the population.

## Conclusion

We found no evidence for a positive health impact of the new activities with an explicit public health focus that were implemented in the Dutch District Approach. Specific attention for network management and the integration of such activities in the wider programme, based on a comprehensive problem analysis, as well as an allocated budget might be needed in order to sort a health impact.

## Supporting information

S1 File Details of analysis. The file describes details on the Nearest Neighbor matching procedure, the stratification procedure, and the estimation of the treatment effect.
(PDF)
