# Supplementary material for: Do inhabitants profit from integrating a public health focus in urban renewal programmes? A Dutch case study
Source: PLoS One. 2022 Jun 24;17(6):e0270367. doi: 10.1371/journal.pone.0270367 (PMC9232143; doi:10.1371/journal.pone.0270367)
Supplement: S1 File — The file describes details on the Nearest Neighbor matching procedure, the stratification procedure, and the estimation of the treatment effect. (PDF) [file pone.0270367.s001.pdf]

S1 Details of analysis

In our study, confounding could exist on two levels: at the neighbourhood level, where living conditions may differ between districts, and at the individual level, where the demographic composition of individuals may vary between districts. Following [10], we deal with the confounding by using a nearest neighbour matching on neighbourhood characteristics and stratification on individual characteristics, respectively. We summarize this approach below.

Stage 1: Nearest neighbour matching on neighbourhood characteristics.

Using information on their physical and social neighbourhood characteristics and safety, as measured at January 1st 2008, we matched the HDE target districts with non-HDE target district on postal code level using nearest neighbour matching. Given the small number of neighbourhoods, we used simple 1:1 matching with Mahalanobis distance measure. The matching eventually led to a selection of 34 non-HDE districts (which encompassed 34 postal codes in total). The matching led to a modest improvement in balance (see table below).

Table The balance for the match between HDE target and non-HDE target

	Before matching			After matching		
	Means treatment	Means control	Mean difference	Means treatment	Means control	Mean difference
% compact housing	0.325	0.333	-0.008	0.325	0.334	-0.008
% apartment	0.694	0.706	-0.012	0.694	0.702	-0.008
% old housing	0.724	0.708	0.016	0.724	0.712	0.013
% social housing	0.644	0.569	0.075	0.644	0.583	0.061
No. houses	4385.588	4612.958	-254.370	4385.588	4463.353	-104.765
No. withdrawals	79.000	62.104	16.896	79.000	43.971	35.029
% satisfied with residence	72.898	74.933	-2.035	72.898	73.973	-1.075
% satisfied with surroundings	64.548	63.233	1.315	64.548	63.840	0.708
% graffiti	31.990	33.329	-1.339	31.990	32.357	-0.367
% vandalism	41.187	34.474	6.714	41.187	34.243	6.944
% nuisance neighbours	28.267	31.098	-2.831	28.267	29.547	-1.279
% nuisance residents	36.492	40.193	-3.701	36.492	39.062	-2.569
% fear of being robbed or bothered	35.414	33.979	1.435	35.414	33.140	2.274

Stage 2: Stratification on individual characteristics.

Within the selected districts, we further used exact stratification on individual characteristics. The following 5 characteristics were considered: age (3 groups), sex, household income (3 groups), the household composition (2 groups) and ethnicity. Full stratification was not feasible due to the limited sample size, thus we considered partial stratification. Following [10], we selected the characteristics that contributed most to the explanation of that particular outcome using the Random Forest algorithm (see table below).

Outcome	Covariate 1	Covariate 2	Covariate 3	Covariate 4

General health	Age	Household income	-	
Smoking	Age	Education	Sex	
BMI	Age	Education	-	
Mental Health	Household income	Age	Sex	
Walking	Age	-	-	
Biking	Ethnicity	Education	Age	
Doing sports	Age	Education	Household income	Ethnicity

Estimating the treatment effect

After assigning observations to their respective strata, we fit a linear regression model on each stratum in which at least two observations are found for each treatment and time:

$$Y_s = \beta_{s,0} + \beta_{s,1} \times Treatment + \beta_{s,2} \times Time + \delta_s Treatment \times Time,$$

where s denotes a stratum, Y_s is the outcome, $Treatment$ is an indicator function (0=target non-HDE and 1=target HDE), and $Time$ a categorical variables (0=2003-2008Q2, 1=2008Q3-2011, 2=2012-2014). $\beta_{s,0}$, $\beta_{s,1}$, $\beta_{s,2}$ and δ_s are the parameters to be estimated, with δ_s being the stratum-specific treatment effect.

The point estimate for the *overall* treatment effect δ can be estimated by taking a weighted estimate

$$E[\delta] = \sum_{s=1}^{S} \frac{N_s}{N} \delta_s$$

With the weights being defined the share of the number individuals N_s in stratum s in the total population size N. The corresponding variance of $E[\delta]$ is then:

$$Var(E[\delta]) = \sum_{s=1}^{S} \left(\frac{N_s}{N}\right)^2 Var(\delta_s)$$
